# Parental effects on offspring sex ratio in the Numbat (*Myrmecobius fasciatus*): does captivity influence paternal sex allocation?

**DOI:** 10.1093/jmammal/gyad067

**Published:** 2023-08-10

**Authors:** Renée C Firman, Connor M Ellis, Sian Thorn, Peter R Mawson

**Affiliations:** Centre for Evolutionary Biology, School of Biological Sciences, University of Western Australia, 35 Stirling Hwy, Crawley, Western Australia 6009, Australia; School of Biological Sciences, University of Western Australia, 35 Stirling Hwy, Crawley, Western Australia 6009, Australia; Centre for Evolutionary Biology, School of Biological Sciences, University of Western Australia, 35 Stirling Hwy, Crawley, Western Australia 6009, Australia; School of Biological Sciences, University of Western Australia, 35 Stirling Hwy, Crawley, Western Australia 6009, Australia; Centre for Evolutionary Biology, School of Biological Sciences, University of Western Australia, 35 Stirling Hwy, Crawley, Western Australia 6009, Australia; School of Biological Sciences, University of Western Australia, 35 Stirling Hwy, Crawley, Western Australia 6009, Australia; Perth Zoo, Department of Biodiversity, Conservation and Attractions, 20 Labouchere Road, South Perth, Western Australia 6151, Australia

**Keywords:** ex situ conservation, male-driven sex allocation, marsupial, *Myrmecobius fasciatus*, offspring sex ratio, population viability analysis

## Abstract

Sex allocation theories predict that under different ecological conditions the production of sons and daughters will affect parental fitness differently. Skewed offspring sex ratios often occur under captive conditions where individuals are exposed to nutritional and social conditions that differ from nature. Here, we analyzed 29 years of offspring sex ratio data from a captive population of an endangered marsupial, the Numbat (*Myrmecobius fasciatus*). We partitioned variation in offspring sex ratio based on parental origin (captive- vs. wild-bred), parental weight, maternal age, and maternal reproductive history. Our analyses revealed no effect of parental weight or maternal origin on offspring sex ratio—however, there was a significant effect of paternal origin. Data visualization indicated that captive-bred males tended to produce male-biased litters. We discuss the result in relation to recent studies that have shown that male mammals have the capacity to be arbiters of sex allocation and highlight candidate mechanisms, but consider it with caution due to the small sample size from which the result was derived. We performed a population viability analysis (PVA) to explore the potential impact of a sex ratio skew on the sustainability of the captive Numbat population under hypothetical scenarios. Our PVA revealed that supplementation with wild individuals is critical to the persistence of the captive Numbat population and that a biased sex ratio will lead to extinction of the captive colony under certain conditions. Overall, our study demonstrates that covert sex ratio skews can persist undetected in captive populations, which have the potential to become impactful and compromise population sustainability under changed management processes.

## Teaser

We explored whether captivity influences offspring sex ratios in a critically endangered marsupial, the Numbat. We found that captivity does not influence how mothers allocate sex, but we did uncover an intriguing pattern that, relative to wild males, captive born fathers produced more sons than daughters. Our study highlights the potential for covert sex ratio skews to persist undetected in captive breeding programs.

Many species have been reported to bias offspring sex ratios, but deviations from parity have been difficult to understand and are often inconsistent (Cockburn et al. 2002; Navara 2018). Evolutionary theory predicts a number of scenarios where parents should bias their investment to one sex over the other (West 2009). That is, when fitness returns are sex-specific, selection should favor the facultative adjustment of offspring sex ratios to the sex with the highest inclusive fitness return (Fisher 1930; Hamilton 1967; Trivers and Willard 1973; Clark 1978; Burley 1981). Theories of sex allocation predict that daughters will be favored when there is intense competition among brothers for mates (Hamilton 1967), or that sons will be favored when mothers are in good condition (Trivers et al. 1973) or when females are mating with attractive (Burley 1981) or fertile (Gomendio et al. 2006) males. Other theories suggest that the dispersing (Clark 1978) or philopatric (Pen and Weissing 2000) sex should be favored under certain circumstances. Research on sex allocation in mammals has largely focused on female traits, with offspring sex ratio variation having been investigated in the context of maternal condition (often proxied by age), reproductive experience, social rank, nutritional status, or level of stress (e.g., Kojola and Eloranta 1989; Saltz 2001; Brown and Silk 2002; Lindström et al. 2002; Schwanz and Robert 2014; Moore et al. 2015; Fishman et al. 2018; Firman 2020). However, fathers will also benefit from producing more of the sex with the greatest fitness return (Edwards and Cameron 2014; Douhard 2018; Douhard and Geffory 2021) and recent studies have suggested that males may influence offspring sex, either directly or indirectly, in an adaptive way (Gomendio et al. 2006; Saragusty et al. 2012; Douhard et al. 2016; Malo et al. 2017; Perret 2018; Edwards et al. 2019; Lavoie et al. 2019; Firman et al. 2020).

Captive populations have proven to be useful for studying sex allocation because, by design, they often control or eliminate factors that influence sex allocation, for example, variation in nutrition, while facilitating the testing of others, such as the parental effects of age, that is, through known pedigrees (Mace 1990; Vermeer and Devreese 2015; Tanaka et al. 2019); origin, that is, captive- versus wild-born; (Dennis et al. 2007); translocation, that is, wild to captivity (Linklater 2007); social rank (Nevison et al. 1996; Perret 2018), and stress (Moore et al. 2015; Firman 2020; Martin et al. 2020). Ex situ conservation strategies, such as captive breeding for reintroduction and population augmentation, are useful tools to help recover threatened species (Bowkett 2009; Conde et al. 2011). Not without criticism (e.g., high costs, low success rates; Fischer and Lindenmayer 2000), ex situ conservation programs operate under a complex management process that typically connects genetic and demographic analyses with scientifically based husbandry protocols to guide each population toward a target size that will ensure its continued survival (Faust and Thompson 2000; Mawson and Lambert 2017). Ex situ populations are typically small, so even slight deviations from sex ratio parity can negatively impact population viability and sustainability. Indeed, an overproduction of one sex can hinder conservation efforts by compromising not only the maintenance of the captive population itself but also, critically, growth of the remaining natural or reintroduced populations that captive-bred individuals seed (Robertson et al. 2006).

**Fig. 1.**
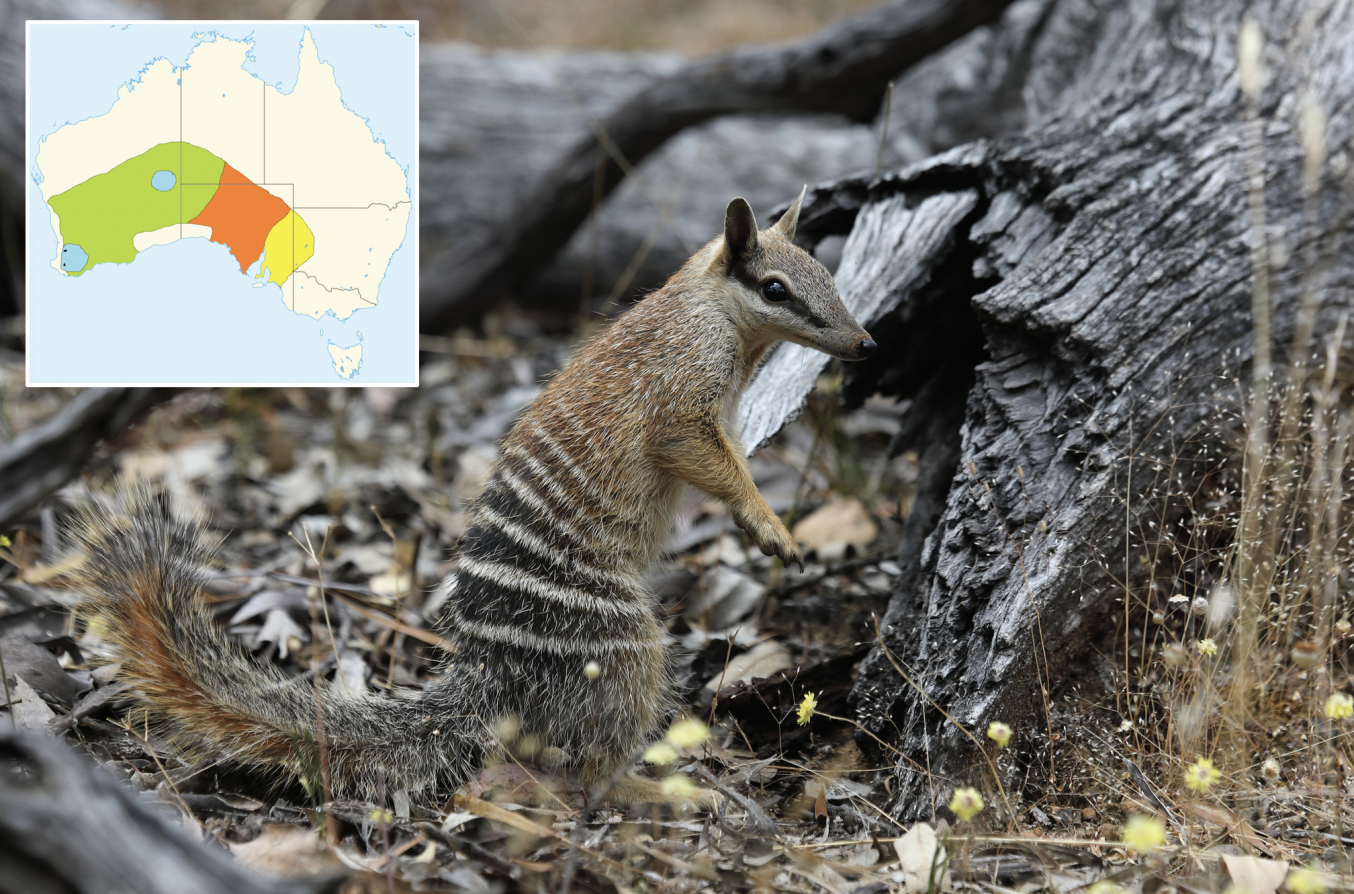
A wild Numbat (*Myrmecobius fasciatus*) of the Dryandra population in Western Australia (credit: Rob McLean). The Numbat is described as being a medium-sized marsupial (length = 35–45 cm; weight = 300–715 g) with a conspicuous black stripe running from the tip of the muzzle through the eyes to the base of the small, round-tipped ears and characteristic white stripes across the lower back. Insert: historical range map of the Numbat with extinctions shown in color (1800–1910 = yellow; 1910–1930 = orange; 1930–1960 = green; 1960–1980: blue) and current naturally occurring populations shown as black dots. Source: https://en.wikipedia.org/wiki/Numbat#/media/File:Numbat_historical_map.png, based on map in (Fumagalli et al. 1999).

Captive breeding has become a successful pathway for the reestablishment and/or augmentation of many of Australia’s endangered wild mammal populations (Harley et al. 2018). One ongoing success story involves a shy and elusive marsupial, the Numbat (*Myrmecobius fasciatus*; Fig. 1). Over the past two centuries, the Numbat has suffered a huge reduction in population size and geographical distribution, primarily due to habitat clearing and predation by introduced species; once spanning the southern part of Australia, the species is now restricted to two locations in the southwest of Western Australia (Dryandra Woodland and Upper Warren; Friend 1990; Hayward et al. 2015; Fig. 1). The dramatic population decline and range reduction of the Numbat led to the development of a reintroduction initiative in the 1990s (Dpaw 2017). As part of this initiative, and as a safeguard against the loss of the two remaining wild populations, in 1993 the Perth Zoo (Western Australia) established a breed-for-release program (Mawson et al. 2017; Harley et al. 2018). To date, this program has successfully released 283 individuals into the wild (Harley et al. 2018). The captive Numbat population has also been used as a valuable resource for research projects and has therefore played a critical role in advancing knowledge on an endangered marsupial that is notoriously difficult to study in the wild (Dpaw 2017).

The Perth Zoo Numbat breed-for-release program provides excellent opportunity to data-mine for covert skews in offspring sex ratio. The longevity of the program suggests that the colony is managed effectively and that, generally, sex ratios persist at parity. Still, captive populations can decline rapidly, and subtle sex ratio skews can go undetected and only become evident upon close analysis. Here, we collected data from 101 litters produced by the captive population over a 29-year time period. We partitioned offspring sex ratio variation based on different factors, including parental origin (i.e., those either being sourced from the wild or bred in captivity), maternal age, and maternal reproductive history (i.e., primiparous or multiparous). Due to the breeding design of the colony there was disparity in the number of litters fathered by wild- versus captive-bred males (i.e., five times as many wild-sourced than captive-bred males contributed to the data set)—however, our analyses revealed a significant effect of paternal origin on offspring sex ratio. We interpret this result with caution due to a small sample size, but consider recent research that has demonstrated that experiences during early development can influence mechanisms of paternal sex allocation (Lavoie et al. 2019; Firman et al. 2020). We then used population viability analysis (PVA) to explore how a sex ratio skew would influence the captive Numbat population under different scenarios of supplementation and with an increased carrying capacity. Overall, our investigation demonstrates that subtle sex ratio biases can persist undetected in captive populations and that these may lead to significant, and potentially detrimental, impacts if management options or operational parameters change.

## Materials and Methods

### Captive population.

Detailed information on the spatial and reproductive ecology of numbats in the wild is provided in the Supplementary Data SD1. From 1993 until present day, the Perth Zoo Numbat breed-for-release program has been the primary source of stock animals used for wild reintroductions. Comprehensive husbandry protocols have been developed and optimized by the Perth Zoo (Power and Monaghan 2007; Jose and Power 2019). It is not logistically possible to supply captive numbats with their full daily termite requirement. Therefore, the breeding colony is maintained on a manufactured diet consisting primarily of egg and milk powder (“termite custard”) and supplemented with termites (Power et al. 2007). Numbats are housed in outdoor enclosures that mimic the natural habitat by including hollow logs, climbing logs, bushy shrubs, and open areas (Power et al. 2007). Artificial underground nesting chambers are used to mimic burrows, although females typically dig their own borrows in the sandy-loam substrate (Jose et al. 2019). Adults are housed individually except during the breeding season, when a series of interconnecting enclosures (5 × 3 × 2 m) are used to accommodate breeding pairs. Thus, each breeding pair has access to, and can move freely between, three enclosures (Supplementary Data SD1). Screens are used to visually separate each breeding pair from all other individuals (Power et al. 2007). The production of young is a synchronized and highly seasonal event, both in the wild and captivity, from late December to February (Power et al. 2009; Hogan et al. 2012) and captive-held male numbats undergo the same seasonal changes in sperm production (testes size) and gland secretions (gland size) observed in wild males (Power et al. 2009; Supplementary Data SD1). When first paired with a female, typically a male will mark the enclosure with sternal gland secretion and urine. The male will follow the female, eliciting vocalizations that consist of soft clicks. Males inspect female estrus status by sniffing the cloaca. When estrus is detected the male will attempt to mount the female, at which stage the female may reject or accept the mating (Jose et al. 2019). Mating lasts for a few minutes to an hour and is completed with a deposition of a mating plug (Power et al. 2007). Individuals breed as monogamous pairs and are assigned mates by Zoo personnel (no choice is given). On occasions males may mate with two females in a season, either being paired consecutively with two females or, more rarely, two females at the same time (Power et al. 2007; Jose et al. 2019). Typically, females only produce one litter each year. However, if a first litter is lost within a few weeks of birth, a second litter may be produced (Jose et al. 2019).

Following a 14-day gestation, females produce a litter of typically 3 or 4 embryos (maximum = 4, average = 3.5; Jose et al. 2019). Immediately following parturition, embryos crawl to the pouch and attach to the teat. Offspring size and sex ratio at birth are not measured because external genitalia are not discernible at this early stage of development and to manipulate joeys to acquire this data poses a risk that the mother will abandon the altricial pouch young (Jose et al. 2019). Joeys are nursed in the pouch for 170–200 days before being deposited in a burrow (until late July) where they will continue to suckle for a further ~100 days (Dpaw 2017). It is at the time of burrow deposition that joeys are first sexed and weighed. Joeys are weaned between 10–11 months of age (Jose et al. 2019). Some captive-bred individuals (typically females) are retained within the colony and contribute to breeding once they reach sexual maturity (males at 2 years old, females at 1 year old). In addition, wild individuals sourced from the Dryandra Woodland population, and as of 2020 also the Upper Warren population, are routinely brought into captivity and contribute to breeding (Dpaw 2017). As a result, different combinations of wild- and captive-bred mothers and fathers contribute to each generation. Typically, numbats brought in from the wild are captured between late October through to mid-November. Adults are held in quarantine for 30 days and then paired with a suitable mate(s) from late December to late February (Power et al. 2009; Hogan et al. 2012). Wild-caught juvenile males (<2 years old) are maintained in captivity until they are sexually mature, that is, for ~13 months (Jose et al. 2019). Wild-caught individuals rarely fail to settle in captivity, but when they do they are released back to the wild before breeding. Likewise, wild-caught individuals that are poor reproductive performers (i.e., males that fail to inseminate females or females that fail to produce a litter) are usually returned to the wild or, depending on their age, placed with other institutions to be used for educational purposes.

### Data acquisition and statistical analyses.

The Zoological Information Management System (ZIMS; managed by Species360) is a centralized web-based information system used by zoological institutions worldwide to pool life history, behavior, and health data and facilitate animal husbandry, health, and breeding management processes. We used the ZIMS data set from numbats held at Perth Zoo to assign parentage to litters, calculate offspring sex ratios, and perform analyses based on parental origin (Supplementary Data SD2). The data set included a unique identification (ID) code for each individual, as well as the ID and origin of the parents (captive or wild birth). The data set also included the (i) parent IDs, (ii) sex, (iii) date of birth, (iv) body weight, and (v) date of first weighing for each individual offspring. We identified 125 discrete litters born between January 1993 and December 2021. Offspring that died before the first weighing were often not sexed (sex defined as “undetermined” in the ZIMS data set). We excluded the litters with “undetermined” offspring from our data set (*n* = 24), which left a total of 101 litters included in our analyses. Of the 101 litters, 43 and 58 litters were produced by captive-bred (*n* = 23) and wild (*n* = 24) mothers, respectively. Eleven of the 101 litters were sired by captive-bred males (*n* = 6), while 90 litters were sired by males sourced from the wild (*n* = 34). For the offspring survival and weight analyses, we used all available data for offspring that had been assigned a sex and had been weighed around the time of burrow deposition. Age of offspring at first weighing was calculated as the difference between actual birth date and the date of weighing, but for 17 litters the date of birth was only known to between ±1 day to ±7 days. For these 17 litters, we used the midpoint of the range in possible birth dates in our calculation of age at first weighing. We excluded one individual that been hand-reared, showed ill-health and died around the time of weaning, resulting in a total of 316 joeys from 98 litters. Offspring mortality was annotated in the second ZIMs data set as “Died before weighing,” which allowed us to score offspring survival from birth to ~200 days of age.

Linear mixed models (LMMs) and generalized linear mixed models (GLMMs) were conducted in R v.3.5.1 using the *lmer* or *glmer* functions implemented within the package lme4 (R Core Team 2021). LMMs were used to analyze origin-based differences in maternal, paternal, and offspring body mass, as well as the relationship between age and body mass among captive-bred individuals. GLMMs using the *cbind* function were used to analyze origin-based differences in offspring number (family = Poisson), offspring survival (*n*_surviving offspring_/*n*_total offspring number_; family = binomial), and offspring sex ratio (*n*_sons_/*n*_total offspring number_; family = binomial). An additional GLMM was performed to test for sex differences in offspring survival, this time without using the *cbind* function as this approach restricts testing for offspring-level differences (i.e., sex-specific variation). We applied a GLMM with the intercept removed to test for offspring sex ratio parity based on parental origin (i.e., against 0.5; family = binomial). A GLMM was applied to test for effects of maternal age and reproductive history (i.e., primiparous or multiparous) on offspring sex ratio in a reduced data set that included only litters born to mothers of captive origin (i.e., those for which maternal age and reproductive history could be determined; family = binomial). To account for nonindependence, mother ID and/or sire ID were included as random effects in all models. An additional random factor of litter ID was included in the offspring body mass LMM to account for nonindependence of individuals from the same litter. Parental condition has the potential to influence fecundity. Therefore, mother and father body mass were included as covariates in the offspring number GLMM and the offspring body mass LMM (note that maternal/paternal origin was nonsignificant and therefore removed from this model). Offspring sex was also included as a covariate in the offspring body mass LMM. Parental condition may also influence offspring sex ratios. Therefore, mother and father body mass were initially included in the GLMM; however, they were found to be nonsignificant and subsequently removed from the final model. All interaction terms were initially included in the models, but subsequently removed if found to be nonsignificant. The final LMMs and GLMMs are presented in the tables.

### Population viability analysis.

The status and efficiency of management measures for captive populations are often evaluated and graded with the use of modeling, such as PVA (Schwartz et al. 2017; Lacy 2019). Here, we used this tool to explore the potential impact of skewed sex ratios on the viability of the captive Numbat population under different supplementation, harvesting and carrying capacity scenarios. We used *Vortex* version 10.0.7.9 (Lacy and Pollak 2017) to generate baseline and case study PVA models. This software is an individual-based simulation model that simulates stochastic demographic, genetic, and environmental processes from birth to death, throughout each year and for each individual following the parameters and probabilities given by the user (Lacy et al. 2017). Each scenario was simulated 1,000 times, using a 1-year time step over a period of a 100 years to allow long-term population trends to be observed and evaluated. The default order of events in each time step was used. Population extinction was defined as occurring when one sex remained. We ran a number of case study scenarios where the baseline model was re-fitted and ran with different combinations of an equal (0.5) or skewed sex ratio (0.7), with or without supplementation and with or without an increased carrying capacity (40 individuals). We provide full details of our models in Supplementary Data SD3 and a summary as: (1) male-biased sex ratio (all other parameters = baseline); (2a) equal sex ratio, no supplementation; (2b) male-biased sex ratio, no supplementation; (3a) increased carrying capacity (all other parameters = baseline); (3b) increased carrying capacity, male-biased sex ratio; (4a) increased carrying capacity, equal sex ratio, no supplementation; and (4b) increased carrying capacity, male-biased sex ratio, no supplementation. We generated standard graphs to visualize the population projections for each of these scenarios.

## Results

**Fig. 2.**
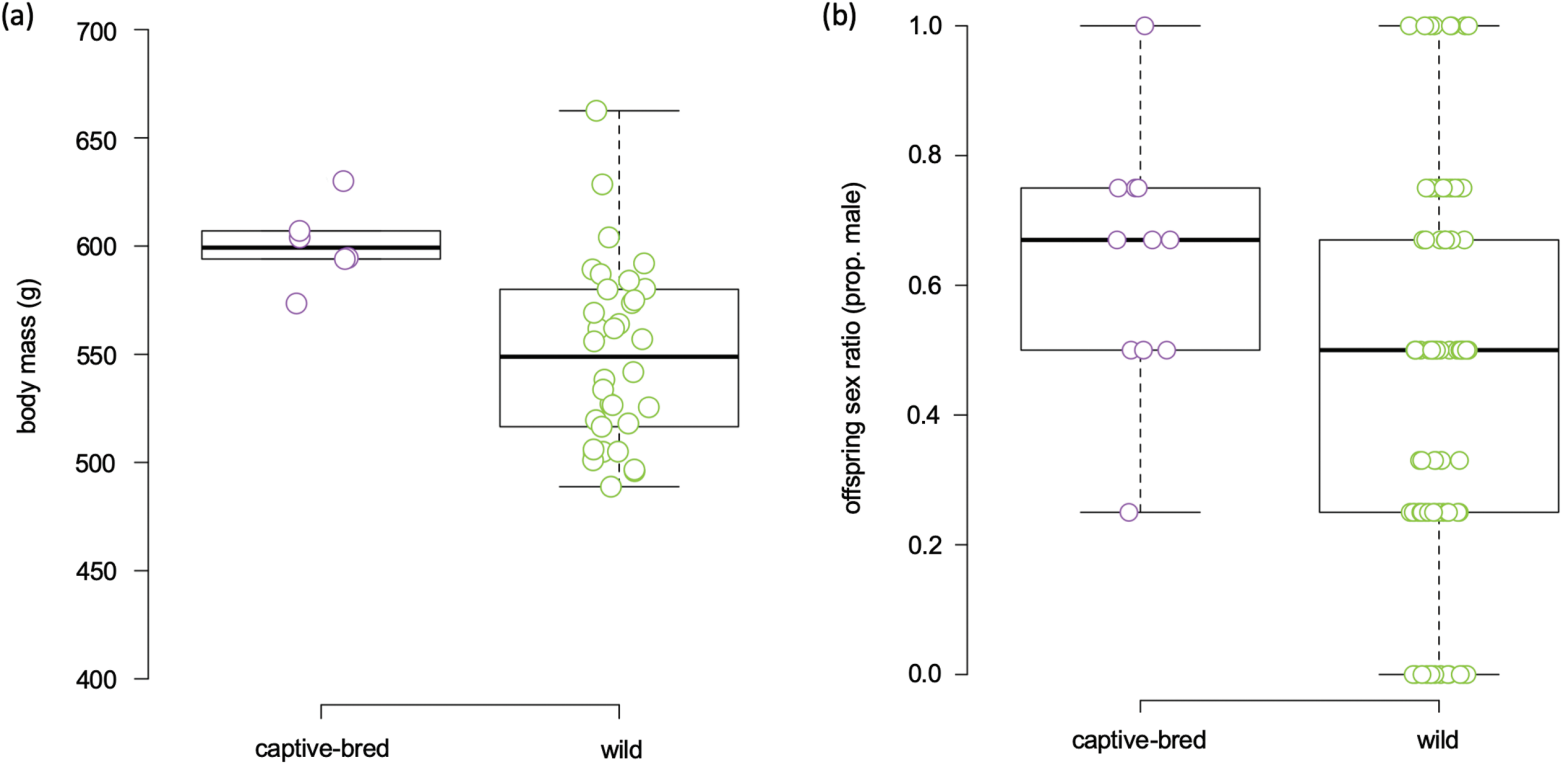
(a) Captive-bred sires (*n* = 6) were, on average, heavier than sires sourced from the wild (*n* = 34). (b) There was a significant effect of paternal origin on offspring sex ratio (OSR) (see Table 2). Visualization of the data suggests that captive-bred males produce more sons (median OSR = 0.7; although does not differ from 0.5; see Table 1) compared to sires sourced from the wild (median OSR = 0.5).

There was a significant relationship between age and body mass among captive-bred mothers and fathers (Table 1, Supplementary Data SD4). Our analyses revealed no origin-based difference in maternal body mass, but there was a significant effect of origin on paternal body mass; captive-bred sires were on average heavier than sires born in the wild (Table 1, Fig. 2a). Parental origin did not influence offspring number (Table 1) and there was no relationship between offspring number and offspring sex ratio (Table 2). There were no sex-based or paternal effects on offspring survival (Table 2). However, our analysis revealed that there was an effect of maternal weight on offspring survival with reduced survival among offspring born to heavier mothers (Table 2, Supplementary Data SD3). There was also a nonsignificant trend of greater survival to an age of ~200 days among offspring born to wild-sourced mothers (mean ± *SE* prop. litter surviving: 0.76 ± 0.05) compared to captive-bred mothers (0.56 ± 0.06; Table 2, Supplementary Data SD3). After accounting for variation due to offspring age, the reduced LMM analyzing offspring body mass (i.e., with parental origin removed) revealed no overall effect of either mother or father body mass. However, there was a significant interaction between mother and father body mass on offspring mass, whereby larger mothers, but smaller fathers, produced larger offspring (Table 1; and see figure in Supplementary Data SD3). There was also a tendency for female offspring (*n* = 164) to be heavier than male offspring (*n* = 152), although this effect was not significant (females = 187.6 ± 5.1 g, males = 181.9 ± 5.6 g; Table 1).

**Table 1.** Linear mixed models (LMMs) investigating the effect of birth origin (captive, wild) on maternal and paternal body mass in the Perth Zoo captive Numbat population. LMMs testing maternal and paternal age on body mass is also included (captive individuals only). An LMM including age, sex, and maternal and paternal body mass was applied to test for variation in offspring body mass. “Wild” and “male” were the reference levels where relevant. The *P*-values in bold are significant at <0.05.

Fixed effects	Estimate	±*SE*	Type II, Wald χ^2^	*P*-value
(a) Maternal body mass				
Intercept	474.538	12.372		
Origin	8.314	16.690	0.248	0.618
(b) Maternal body mass (captive mothers only)				
Intercept	400.330	14.840		
Maternal age	35.700	5.340	44.690	**<0.001**
(c) Paternal body mass				
Intercept	601.500	18.350		
Origin	−50.330	19.730	6.506	**0.011**
(d) Paternal body mass (captive fathers only)				
Intercept	547.950	17.222		
Paternal age	16.556	4.744	12.182	**<0.001**
(e) Offspring body mass				
Intercept	−1,1171.000	316.600		
Age	2.031	0.156	168.673	**<0.001**
Dam weight	1.712	0.646	1.291	0.256
Sire weight	1.565	0.556	0.328	0.567
Dam weight × sire weight	−0.003	0.001	7.628	**0.006**
Sex	4.752	2.701	3.095	0.079

**Table 2.** Generalized linear mixed models (GLMMs) investigating factors influencing offspring number (family = Poisson) and sex ratio (family = binomial) in the Perth Zoo captive Numbat population. “Male,” “wild,” and “primiparous” were the reference levels where relevant. GLMMs with the intercept removed to test for offspring sex ratio parity based on paternal origin (i.e., against 0.5) are included (f). The *P*-values in bold are significant at <0.05.

Fixed effects	Estimate	±*SE*	Statistic*	*P*-value
(a) Offspring number				
Intercept	0.692	0.791		
Maternal origin	−0.004	0.111	0.001	0.971
Paternal origin	−0.078	0.183	0.182	0.669
Maternal body mass	0.001	0.001	1.302	0.254
Paternal body mass	<0.001	0.001	0.035	0.852
(b) Offspring survival: sex				
Intercept	9.410	1.384		
Sex	−0.012	0.758	<0.001	0.988
(c) Offspring survival: parental effects				
Intercept	−0.260	0.998		
Maternal origin	0.269	0.152	3.130	0.077
Paternal origin	−0.001	0.255	<0.001	0.999
Maternal weight	−0.002	0.001	4.213	**0.040**
Paternal weight	0.002	0.001	1.150	0.283
(d) Sex ratio				
Intercept	0.137	0.560		
Offspring number	−0.066	0.152	0.187	0.665
(e) Sex ratio				
Intercept	−0.638	0.362		
Maternal origin	0.094	0.223	0.178	0.673
Paternal origin	0.774	0.354	4.785	**0.029**
(f) Sex ratio: paternal origin				
Captive	−0.580	0.334	−1.737	0.082
Wild	0.190	0.117	1.625	0.104
(g) Sex ratio (captive mothers only)				
Intercept	0.252	0.494		
Maternal age	−0.048	0.142	0.113	0.737
Reproductive history	−0.157	0.397	0.155	0.694

*a, f: *z*-value; b–e, g: Type II Wald, χ^2^.

Our analysis revealed that paternal origin had a significant effect on offspring sex ratio, while maternal origin did not (Table 2, Fig. 2b, Supplementary Data SD3). Visualization of the data revealed that this effect was attributable to a biased production of sons among the 11 litters born to the six captive-bred males (median OSR = 0.67; although this bias did not differ significantly from parity; Table 2). Maternal age and reproductive history did not influence offspring sex ratios among litters produced by known-age, captive-bred females (Table 2).

**Fig. 3.**
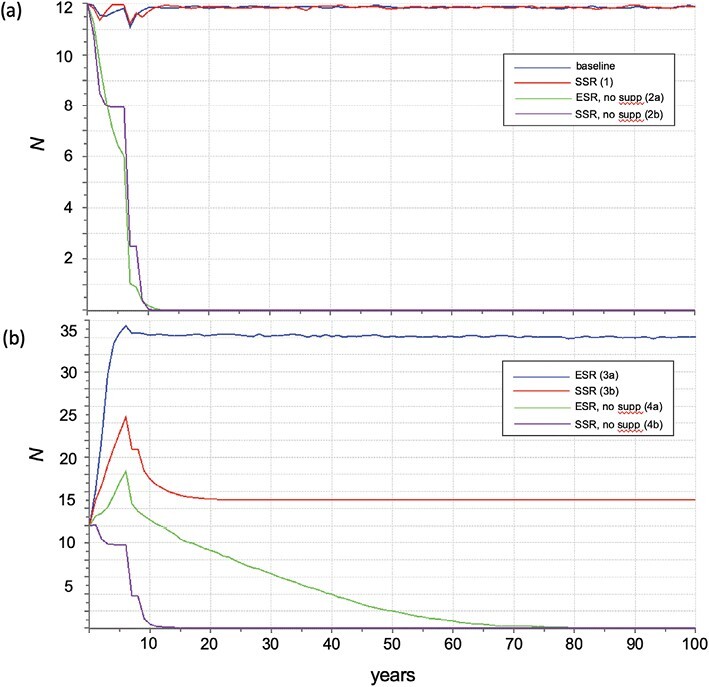
Population viability analysis (PVA) projections of number of individuals (*N*) over time corresponding to a baseline and simulated scenarios for the captive Numbat population. Scenarios are: (a) baseline (blue); scenario 1 = male-biased sex ratio, all other parameters = baseline (red); scenario 2a = equal sex ratio, no supplementation (green); scenario 2b = male-biased sex ratio, no supplementation (purple); (b) scenario 3a = increased carrying capacity, all other parameters = baseline (blue); scenario 3b = increased carrying capacity, male-biased sex ratio (red); scenario 4a = increased carrying capacity, equal sex ratio, no supplementation (green); scenario 4b = increased carrying capacity, male-biased sex ratio, no supplementation (purple).

We used PVA to explore how a male-biased sex ratio skew would influence the persistence of the captive Numbat population under different scenarios. Our baseline PVA demonstrated that the population will continue to persist under the current operational parameters irrespective of whether there is an equal (0.5) or male-biased (0.7) sex ratio (Fig. 3a). However, if supplementation was to cease, then the population crashes within 12 years under an equal sex ratio and 2 years earlier under a skewed sex ratio (Fig. 3b). It is evident that skewed sex ratio would negatively impact population growth, and restrict it from reaching a larger carrying capacity, which is achievable under an equal sex ratio (Fig. 3b). Finally, at sex ratio parity it is predicted that a larger carrying capacity would allow the population to persist for ~70 years if supplementation ceased (Fig. 3b). This is different to what would happen under a skewed sex ratio where the population is predicted to cease at the 12-year mark (Fig. 3b).

## Discussion

Despite the expectation that frequency-dependent selection maintains offspring sex ratios around parity over evolutionary time (Fisher 1930), skewed offspring sex ratios have been documented in all vertebrate classes over ecological time periods, including those with chromosomal sex determination (Navara 2018). Theories propose that this variation may reflect adaptive processes that provide a parental fitness advantage (Hamilton 1967; Trivers et al. 1973; Clark 1978; Burley 1981). Identifying processes of sex allocation in wild populations can be challenging due to the complexity of life histories, environmental variation, and rapidly changing population structures and/or demographics. Studying captive populations, which are mostly buffered from environmental and resource fluctuations and population structure is relatively stable, is one way to address this challenge (Mace 1990; Nevison et al. 1996; Dennis et al. 2007; Linklater 2007; Moore et al. 2015; Vermeer et al. 2015; Perret 2018; Tanaka et al. 2019; Firman 2020; Martin et al. 2020). In this investigation, we extracted and analyzed data from a captive Numbat population that has been managed and maintained at the Perth Zoo for nearly three decades. In this species, females are able to participate in breeding from one year of age (i.e., the breeding season following their birth), while males are unable to breed until 2 years of age. The difference in age of sexual maturity, and the restricted space that is available for holding juveniles while they mature, means that the colony is managed most effectively by preferentially retaining captive-bred females over captive-bred males and primarily sourcing fathers from the wild. As a result, there was disparity in the number of litters fathered by wild- versus captive-bred males. Still, our analysis revealed a significant effect of paternal origin on offspring sex ratio. Visualization of the data indicated that this result was driven by a tendency for captive-bred fathers to produce male-biased litters.

The effect that we observed in the small subset of captive-bred male numbats is intriguing in light of new discoveries in the field of paternal sex allocation and recent research that has revealed that mammalian fathers may play a key role in controlling the sex of their offspring (Edwards et al. 2014; Douhard 2018; Douhard et al. 2021). These paternal effects may be indirect, for example, under the prediction of the mate attractiveness or quality hypothesis that posits that females breeding with attractive or high-quality males should give birth to more sons (Burley 1981; Douhard 2018). Although this hypothesis has mainly been tested using birds (Booksmythe et al. 2017), there is supporting evidence for mammals where paternal quality has correlated with offspring sex ratios at or soon after birth (Saether et al. 2004; Gomendio et al. 2006; Roed et al. 2007; Helle et al. 2008; Douhard et al. 2016; Malo et al. 2017; Tanaka et al. 2019; but see Ozoga and Verme 1985; Small and Smith 1985; Polak et al. 2015; Santos et al. 2015). These studies applied different indices to estimate paternal quality, including age, social rank, fertility, body mass and condition, reproductive success, and inbreeding level (Douhard 2018). There have been no targeted assessments of origin-based variation in male Numbat “quality,” although a study on translocated individuals reported no difference in short-term survival rate between captive- versus wild-bred individuals of either sex (Palmer et al. 2020). Here, our analysis showed that captive-bred fathers were, on average, heavier than fathers sourced from the wild. However, paternal body mass did not explain variation in offspring sex ratio in either the complete data set (captive-bred and wild-sourced fathers), nor in the data subset that included only captive-bred fathers. Similarly, paternal age was not related to offspring sex ratios in our small sample of litters sired by captive-bred males. Thus, we are currently unable to draw any firm conclusions about the degree to which female numbats perceive male quality at the time of mating and whether they bias offspring sex accordingly. Certainly, the prolific swelling of the sternal gland and its use for scent-marking during the breeding season has the potential to serve as an informative secondary sexual trait that signals to females male territory-holding ability and, consequently, male quality (Salamon et al. 1999).

It is possible that the captive-bred father effect on offspring sex ratios is directly male-driven, for example, through variation in the relative number or performance of X- and Y-chromosome-bearing sperm (CBS) that males produce (Gomendio et al. 2007; Saragusty et al. 2012; Malo et al. 2017; Lavoie et al. 2019; Firman et al. 2020). Studies on disparate mammal species, including the Pygmy Hippopotamus (*Choeropsis liberiensis*; Saragusty et al. 2012), the White-footed Deermouse (*Peromyscus leucopus*; Malo et al. 2017), and another Australian marsupial, the Tammar Wallaby (*Notamacropus eugenii*; Edwards et al. 2019), have implicated fathers to be arbiters of sex allocation by reporting positive correlations between sperm (i.e., proportion of X- to Y-CBS) and offspring sex ratios. Specifically, the tammar wallaby study reported a population-level bias in both the production of Y-CBS and the birth of sons (Edwards et al. 2019). Experiments using house mice have demonstrated how differential exposure of males to the scents of future reproductive rivals during early development can influence sperm sex ratios at sexual maturity (Lavoie et al. 2019; Firman et al. 2020), which may be relevant to the captive Numbat population that resides in a different social environment relative to those experienced by wild individuals. Although numbats live a solitary existence in nature, it is inevitable that juvenile males encounter the scent marks of sexually mature males at different times, including while foraging from an early age or during sexual development as they vie to establish a territory of their own. This seems especially true for the breeding season when reproductive males are ranging widely in search of mates (Dpaw 2017). If the captive social environment, where individuals are isolated during development, equates to little exposure to conspecifics scents it may be considered to be relatively benign compared to what males experience in nature. In this sense, a directional effect on sperm sex ratios, and a biased production of sons in an environment where male density is low (i.e., low risk of male–male competition), could be considered to be adaptive (Hamilton 1967). Alternatively, the close, sustained proximity of the males in the captive population (often occupying an enclosure only one removed from the next male) might mean that these males experience increased exposure to “rival” males and their scents relative to males in the wild. Research on different mammal species has indicated that exposure to rivals can elevate testosterone levels (Firman et al. 2020) and that high testosterone level leads to the production of sons (James 1996). However, whether the mechanistic link between these findings is attributable to variation in sperm sex ratios, and whether this accounts for the result we observed here, is yet to be determined.

Nonsperm constituents within the ejaculate have also been implicated in influencing offspring sex ratios. For example, seminal proteins may differentially impact the performance of X- and Y-CBS (e.g., swimming ability) and lead to variation in the fertilization success of male- and female-producing sperm (Gomendio et al. 2007), or differences in seminal glucose levels may differentially affect the survival of male and female conceptus (Edwards and Cameron 2017; Douhard et al. 2021). While these suppositions remain to be tested they are interesting in relation to the captive-bred father effect observed in this study and the possibility that nutritional variation during development (i.e., termite custard vs. wild diet) influences Numbat ejaculate traits as observed in other species (Bunning et al. 2015; Solon-Biet et al. 2015).

We used PVA to examine the effects of a skewed sex ratio on the future of the captive Numbat population under different scenarios, including with or without supplementation and with an increased carrying capacity. We chose to simulate our PVAs using a male-biased sex ratio, equivalent to what we observed among captive-bred males. Our simulations revealed that supplementation is critical to the sustainability of the population regardless of offspring sex ratio. The limited space available to house captive individuals (maximum carrying capacity = 12) means that the genetic viability and sustainability of the population relies heavily on supplementation by wild individuals. If supplementation was to suddenly cease, for example, if the source population suffered a compromising loss and was deemed too vulnerable to seed the ex situ program, the captive population crashes at the 12-year mark under an equal sex ratio and 2 years sooner under a skewed sex ratio. If it was necessary to act and ensure that greater space and resources were invested in supporting a larger captive population (carrying capacity = 40), then the detriment of a skewed sex ratio is most evident. Under a skewed sex ratio, the captive colony fails to reach carrying capacity even when still being supplemented by one wild female and two wild males per year, and stabilizes around *n* = 15. Note that this is an overestimation of the impact, given that wild-sourced sires are expected to produce offspring sex ratios at parity. The most significant impact of a male-biased sex ratio is when the carrying capacity is increased and supplementation ceases. In this situation, a population with an equal sex ratio persists for up to 70 years, whereas a male-biased sex ratio of 0.7 persists for only 12 years. These hypothetical scenarios reveal the potential importance of sex ratio in a return on investment by increased capacity of the captive population.

Although female mammals have opportunities to manipulate the sex of their offspring at multiple levels, from differential sperm “use” to preferential lactation (Robert and Schwanz 2011; Robert and Braun 2012; Quesnel et al. 2017; Navara 2018), our analyses revealed no maternal effects of origin, weight, age, or reproductive history on offspring sex ratio. This result could indicate a true absence of maternal sex-biasing mechanisms in this species. However, limitations of our investigation—for example, the advanced age at which offspring were first sexed and the exclusion of litters that contained unsexed offspring—may have meant that biases in the fertilization (primary) or birth (secondary) sex ratios went undetected. Certainly, to avoid wasted reproductive investment in the unwanted sex, biasing mechanisms are expected to operate during early development (Davison and Ward 1998; Douhard et al. 2016; Firman 2020). A study on the Agile Antechinus (*Antechinus agilis*) revealed that an overabundance of female offspring was evident at multiple postnatal levels (e.g., at birth and among pouch young), suggesting that the strongly female-biased offspring sex ratio skews were attributable to a prenatal mechanism or mechanism(s) (Davison et al. 1998). Sperm assortment and differential fertilization are likely candidates in marsupials that bear sperm crypts and store sperm for up to a few days prior to ovulation, as observed in antechinus (Davison et al. 1998; Robert et al. 2011). Whether such mechanisms operate in numbats is yet to be determined.

Our study demonstrates the potential for an origin-based male-driven offspring sex ratio skew to impact the sustainability of small captive populations and the effectiveness of investing in increased carrying capacity of these populations, with or without supplementation from wild sources. Due to the way in which the Numbat population is managed, and because it is mostly wild-sourced males that contribute to breeding, the observed effect was only among a small number of males. Although our small sample size may shed some doubt on the generality of the result, this outcome is reassuring regarding the successful operation of the breed-for-release conservation program and the potential for this bias to persist among captive-bred fathers. There appears to be no opportunity for this skew to detrimentally impact the population under the current management regime. Future research is required to gauge the generality of, and factors contributing to, paternal sex allocation in marsupials (Edwards et al. 2019). More specifically, because males shed sperm in the urine during the mating season, future work on numbats could test for a direct relationship between sperm and offspring sex ratios in both captive-bred and wild-sourced fathers. Indeed, many components of marsupial biology are advantageous in experimental studies that examine both adaptive and mechanistic hypotheses of mammalian sex allocation (Robert et al. 2011).

## Supplementary Material

gyad067_suppl_Supplementary_Data_SD1

gyad067_suppl_Supplementary_Data_SD2

gyad067_suppl_Supplementary_Data_SD3

gyad067_suppl_Supplementary_Data_SD4

## Data Availability

Available in the Supplementary Data.

## Supplementary Data

**Supplementary Data SD1.**—Study species: spatial and reproductive ecology in nature.

**Supplementary Data SD2**.—Data.

**Supplementary Data SD3**.—Population viability analysis (PVA).

**Supplementary Data SD4**.—Supplementary figures.
