# Supplementary material for: Parental effects on offspring sex ratio in the Numbat (Myrmecobius fasciatus): does captivity influence paternal sex allocation?
Source: J Mammal. 2023 Aug 10;104(5):1036–46. doi: 10.1093/jmammal/gyad067 (PMC10682968; doi:10.1093/jmammal/gyad067)

**Study species: spatial and reproductive ecology in nature**

The numbat is a unique Australian mammal species; it feeds on termites, consuming 15,000-20,000 per day (equating to ~10% of the adult body weight) (Friend 1995), and consequently it is exclusively diurnal in activity (Christensen, Maisey, and Perry 1984). Numbats are solitary animals that occupy exclusive home ranges. Male home ranges are typically larger than female home ranges (Christensen, Maisey, and Perry 1984; DPaW 2017; Hayward et al. 2015) and habitat use by each sex changes during the year; females contract their area of movement in summer, males in winter (DPaW 2017). The species is defined as having a polygynous mating system as males are known to mate with multiple females within a single season (Power and Monaghan 2007), although it is yet to be determined whether or not females also mate multiply. The production of young is a synchronised and highly seasonal event, both in the wild and captivity (late December to February; (Hogan et al. 2012; Power, Lambert, and Matson 2009)). Female numbats exhibit spontaneous ovulation and are polyoestrous, having two or three oestrous cycles approximately 28 days apart during the breeding season (Hogan et al. 2012). Male numbats undergo an annual cyclic change in testosterone production that results in seasonal changes in sperm production, gland secretion, testes size and accessory gland size (Hogan et al. 2012; Power, Lambert, and Matson 2009). In September, the male sternal gland becomes active to produce an oily secretion and the cloaca swells with the enlargement of the bulbourethral gland (Power, Lambert, and Matson 2009). It is at this time that males begin to move outside their winter home range (DPaW 2017). As the mating season approaches, the testes enlarge and begin to produce sperm, reaching a peak in late December (Power, Lambert, and Matson 2009). By January male numbats are ranging widely and traversing the home ranges of multiple females (DPaW 2017).

**Supplementary Literature Cited**

Christensen, P., K. Maisey, and D. H. Perry. 1984. 'Radiotracking the numbat, *Myrmecobius fasciatus*, in the Perup Forest of Western Australia', *Australian Wildlife Research*, 11: 275-88.

DPaW. 2017. "Numbat (*Myrmecobius fasciatus*) recovery plan." In. Western Australia: Department of Parks and Wildlife.

Friend, J. A. . 1995. 'Numbat *Myrmecobius fasciatus*.' in R. Strahan (ed.), *The Mammals of Australia* (Reed Books: Sydney, Australia).

Hayward, M. W., A. S. L. Poh, J. Cathcart, C. Churcher, J. Bentley, K. Herman, L. Kemp, N. Riessen, P. Scully, C. H. Diong, S. Legge, A. Carter, H. Gibb, and J. A. Friend. 2015. 'Numbat nirvarna: conservation ecology of the endangered numbat (Myrmecobius fasciatus) (Marsupialia: Myrmecobiidae) reintorduced to Scotia and Yookamurra Sanctuaries, Australia', *Australian Journal of Zoology*, 63: 258-69.

Hogan, L. A., A. T. Lisle, L. Valentine, S. D. Johnston, and H. Robertson. 2012. 'Non-invasive monitoring of male and female numbat (*Myrmecobius fasciatus*: Myrmecobiidae) reproductive activity', *Animal Reproduction Science*, 133: 237-45.

Power, V., C. Lambert, and P. Matson. 2009. 'Reproduction of the numbat (*Myrmecobius fasciatus*): observations from a captive breeding program', *Australian Mammalogy*, 31: 25-30.

Power, V., and C. Monaghan. 2007. 'Numbats.' in S. Jackson (ed.), *Australian mammals. Biology and captive management.* (CSIRO Publishing: Collingwood, Australia).

Layout of breeding enclosures:


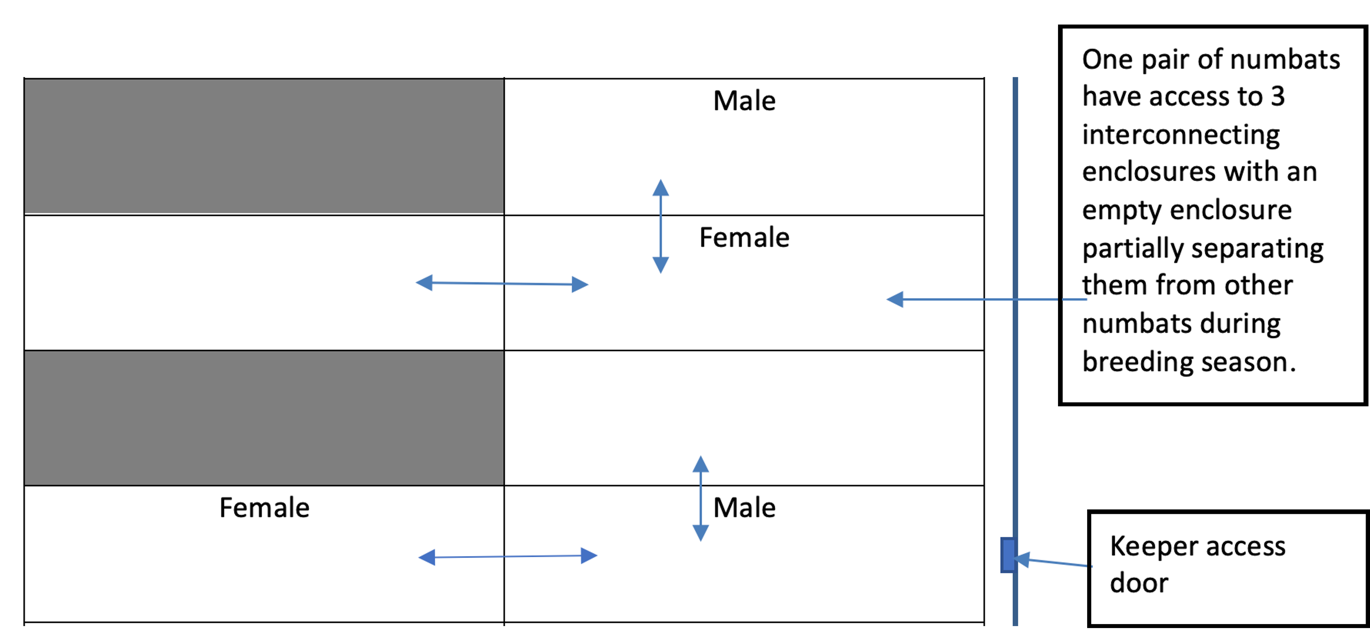

Supplement: gyad067_suppl_Supplementary_Data_SD1 [file gyad067_suppl_supplementary_data_sd1.docx]
