# Supplementary material for: Parental effects on offspring sex ratio in the Numbat (Myrmecobius fasciatus): does captivity influence paternal sex allocation?
Source: J Mammal. 2023 Aug 10;104(5):1036–46. doi: 10.1093/jmammal/gyad067 (PMC10682968; doi:10.1093/jmammal/gyad067)
Supplement: gyad067_suppl_Supplementary_Data_SD3 [file gyad067_suppl_supplementary_data_sd3.docx]

**Population viability analysis (PVA)**

*Baseline scenario*

Basic demographic parameters were used to project the baseline scenario. Inbreeding was modelled according to the default setting (6.29 lethal equivalents, with 50% due to recessive lethal alleles). Environmental variation (EV) concordance of reproduction and survival was not selected due to the captive nature of the population. Reproductive system parameters were obtained from [51] and defined as (i) monogamous; (ii) age of first breeding (years): 1 for females and 2 for males; (iii) maximum age of reproduction (years): 5 for females (that seldom produce young beyond their sixth year) and 8 for males; (iv) maximum lifespan (years): 8 (numbats live to 10 years of age but post-reproductive animals are typically removed from the captive population to avoid expending resources on legacy animals); (v) number of broods per year: 1; (vi) maximum progeny number: 4; and (vii) birth sex ratio: 0.5. The distribution of broods per year were set for 0 broods (12%) and 1 brood (88%) based on the average percentages of mated females that did not (reproductive failure) and did (reproductive success) produce a litter for the period 1997 to 2014. We calculated the proportion of litters that contained one (12%), two (12%), three (20%) and four (56%) joeys and set these values as the distribution of offspring number per female per brood (under reproductive rates). Similarly, we calculated and set early mortality rates from existing offspring survival data (mortality from age 0 to 1: 11/15% females/males). The percentage of males in the breeding pool was set at 100%. The carrying capacity of the captive population was set at its maximum (12 individuals) and the initial population size was established using a specified age distribution (females age 1: 6; males age 2: 6). The number of females (7) and males (8) harvested each year were calculated as the average across an eight-year period (2012–2019). This time period was also used to calculate averages of real supplementation data that were fitted in the model (females after age 1: 1; males after age 2: 2). State variables, catastrophes and genetic information were not included in our PVA. We generated a standard graph to visualise the population projection for this baseline scenario.

*Case study scenarios*

We ran a number of case study scenarios where the baseline model was re-fitted and ran with different combinations of an equal (0.5) or skewed sex ratio (0.7), with or without supplementation and with or without an increased carrying capacity (40 individuals):

1. Male-biased sex ratio (all other parameters = baseline)

2a. Equal sex ratio, no supplementation

2b. Male-biased sex ratio, no supplementation

3a. Increased carrying capacity (all other parameters = baseline)

3b. Increased carrying capacity, male-biased sex ratio

4a. Increased carrying capacity, equal sex ratio, no supplementation

4b. Increased carrying capacity, male-biased sex ratio, no supplementation

In all scenarios the population was harvested because the alternative scenario (unharvested) is unrealistic (i.e., it would not be possible to hold and maintain multiple cohorts of offspring).

|  | **PVA models** | | | | | | | |
| --- | --- | --- | --- | --- | --- | --- | --- | --- |
| **parameters** | **baseline** | **1** | **2a** | **2b** | **3a** | **3b** | **4a** | **4b** |
|  |  |  |  |  |  |  |  |  |
| Species description |  |  |  |  |  |  |  |  |
| inbreeding depression lethal equivalents | 6.29 |  |  |  |  |  |  |  |
| % due to recessive lethals | 50 |  |  |  |  |  |  |  |
|  |  |  |  |  |  |  |  |  |
| Reproductive system |  |  |  |  |  |  |  |  |
| reproductive system | monogamous |  |  |  |  |  |  |  |
| age of first offspring: female | 1 |  |  |  |  |  |  |  |
| age of first offspring: male | 2 |  |  |  |  |  |  |  |
| max. age of reproduction: female | 5 |  |  |  |  |  |  |  |
| max. age of reproduction: male | 8 |  |  |  |  |  |  |  |
| max. lifespan | 8 |  |  |  |  |  |  |  |
| max. # broods/year | 1 |  |  |  |  |  |  |  |
| max. # progeny/year | 4 |  |  |  |  |  |  |  |
| sex ratio at birth | 0.5 | 0.7 | 0.5 | 0.7 | 0.5 | 0.7 | 0.5 | 0.7 |
| density dependent reproduction | no |  |  |  |  |  |  |  |
|  |  |  |  |  |  |  |  |  |
| Reproductive rates |  |  |  |  |  |  |  |  |
| % adult females breeding | 100 |  |  |  |  |  |  |  |
| SD in % breeding due to EV | 10 |  |  |  |  |  |  |  |
| distribution of broods/year: 0 | 12 |  |  |  |  |  |  |  |
| 1 | 88 |  |  |  |  |  |  |  |
| dist. of offspring number/female/brood (%): 1 | 12 |  |  |  |  |  |  |  |
| 2 | 12 |  |  |  |  |  |  |  |
| 3 | 20 |  |  |  |  |  |  |  |
| 4 | 56 |  |  |  |  |  |  |  |
|  |  |  |  |  |  |  |  |  |
| Mortality rates |  |  |  |  |  |  |  |  |
| age 0 to 1: females | 11 |  |  |  |  |  |  |  |
| SD due to EV: females | 10 |  |  |  |  |  |  |  |
| age 0 to 1: males | 15 |  |  |  |  |  |  |  |
| SD to EV: males | 10 |  |  |  |  |  |  |  |
| age 1 to 2: males | 0 |  |  |  |  |  |  |  |
| SD due to EV: males | 0 |  |  |  |  |  |  |  |
|  |  |  |  |  |  |  |  |  |
| Catastrophes |  |  |  |  |  |  |  |  |
| no. of types of catastrophes | 0 |  |  |  |  |  |  |  |
|  |  |  |  |  |  |  |  |  |
| Mate monopolization |  |  |  |  |  |  |  |  |
| % males in breeding pool | 100 |  |  |  |  |  |  |  |
|  |  |  |  |  |  |  |  |  |

A summary of the *Vortex* parameter inputs for the baseline and case study PVA models for the captive numbat population:

Table continued.

|  | **baseline** | **1** | **2a** | **2b** | **3a** | **3b** | **4a** | **4b** |
| --- | --- | --- | --- | --- | --- | --- | --- | --- |

| Initial population size |  |  |  |  |  |  |  |  |
| --- | --- | --- | --- | --- | --- | --- | --- | --- |
| use specified age distribution | yes |  |  |  |  |  |  |  |
| initial population size | 12 |  |  |  |  |  |  |  |
| female age distribution: age 1 | 6 |  |  |  |  |  |  |  |
| male age distribution: age 2 | 6 |  |  |  |  |  |  |  |
|  |  |  |  |  |  |  |  |  |
| Carrying capacity |  |  |  |  |  |  |  |  |
| carrying capacity (K) | 12 |  |  |  | 40 | 40 | 40 | 40 |
| SD om K due to EV | 0 |  |  |  | 0 | 0 | 0 | 0 |
|  |  |  |  |  |  |  |  |  |
| Harvest |  |  |  |  |  |  |  |  |
| population harvested? | yes |  | yes | yes |  |  | yes | yes |
| first year of harvest | 1 |  |  |  |  |  |  |  |
| last year of harvest | 100 |  |  |  |  |  |  |  |
| interval between harvests | 1 |  |  |  |  |  |  |  |
| optional criteria for harvest | 1 |  |  |  |  |  |  |  |
| optional criteria for individuals | 1 |  |  |  |  |  |  |  |
| no. females of each age: 1 | 7 |  |  |  |  |  |  |  |
| no. males of each age: 1 | 8 |  |  |  |  |  |  |  |
| no. males of each age: 2 | 0 |  |  |  |  |  |  |  |
|  |  |  |  |  |  |  |  |  |
| Supplementation |  |  |  |  |  |  |  |  |
| population supplemented? | yes |  | no | no |  |  | no | no |
| first year of supplement | 1 |  |  |  |  |  |  |  |
| last year of supplement | 100 |  |  |  |  |  |  |  |
| interval between supplements | 1 |  |  |  |  |  |  |  |
| optional criteria for supplements | 1 |  |  |  |  |  |  |  |
| female supplement from after age: 1 | 1 |  |  |  |  |  |  |  |
| male supplement from after age: 1 | 0 |  |  |  |  |  |  |  |
| male supplement from after age: 2 | 2 |  |  |  |  |  |  |  |
|  |  |  |  |  |  |  |  |  |
| Genetics |  |  |  |  |  |  |  |  |
| genetic input? | no |  |  |  |  |  |  |  |
|  |  |  |  |  |  |  |  |  |

|  | **PVA models** |
| --- | --- |
