# Supplementary material for: Parental effects on offspring sex ratio in the Numbat (Myrmecobius fasciatus): does captivity influence paternal sex allocation?
Source: J Mammal. 2023 Aug 10;104(5):1036–46. doi: 10.1093/jmammal/gyad067 (PMC10682968; doi:10.1093/jmammal/gyad067)
Supplement: gyad067_suppl_Supplementary_Data_SD4 [file gyad067_suppl_supplementary_data_sd4.docx]

Relationship between parental age and body mass among captive-bred mothers (pink; *n* = 23; *n*_litters_ = 43) and fathers (blue; *n* = 6; *n*_litters_ = 11) that contributed to the Perth Zoo numbat population (1993 – 2021):


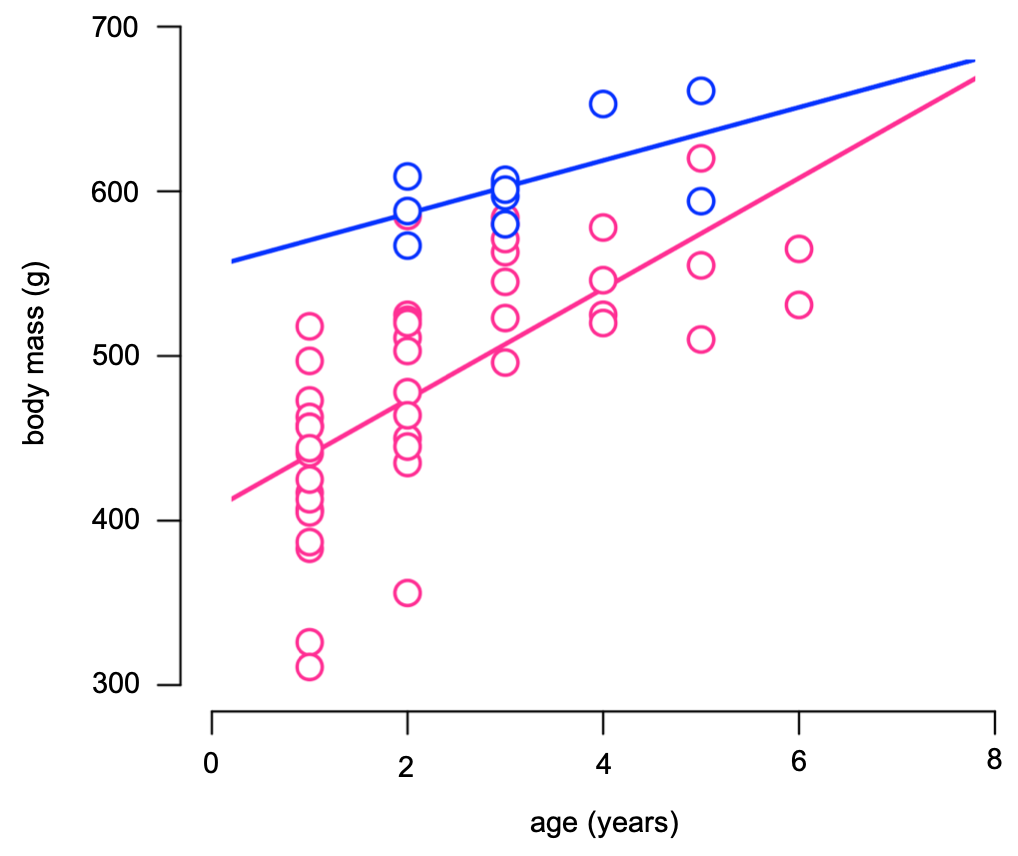


Relationship between (a) maternal body mass and offspring survival and (b) parental body mass and offspring body mass:

**
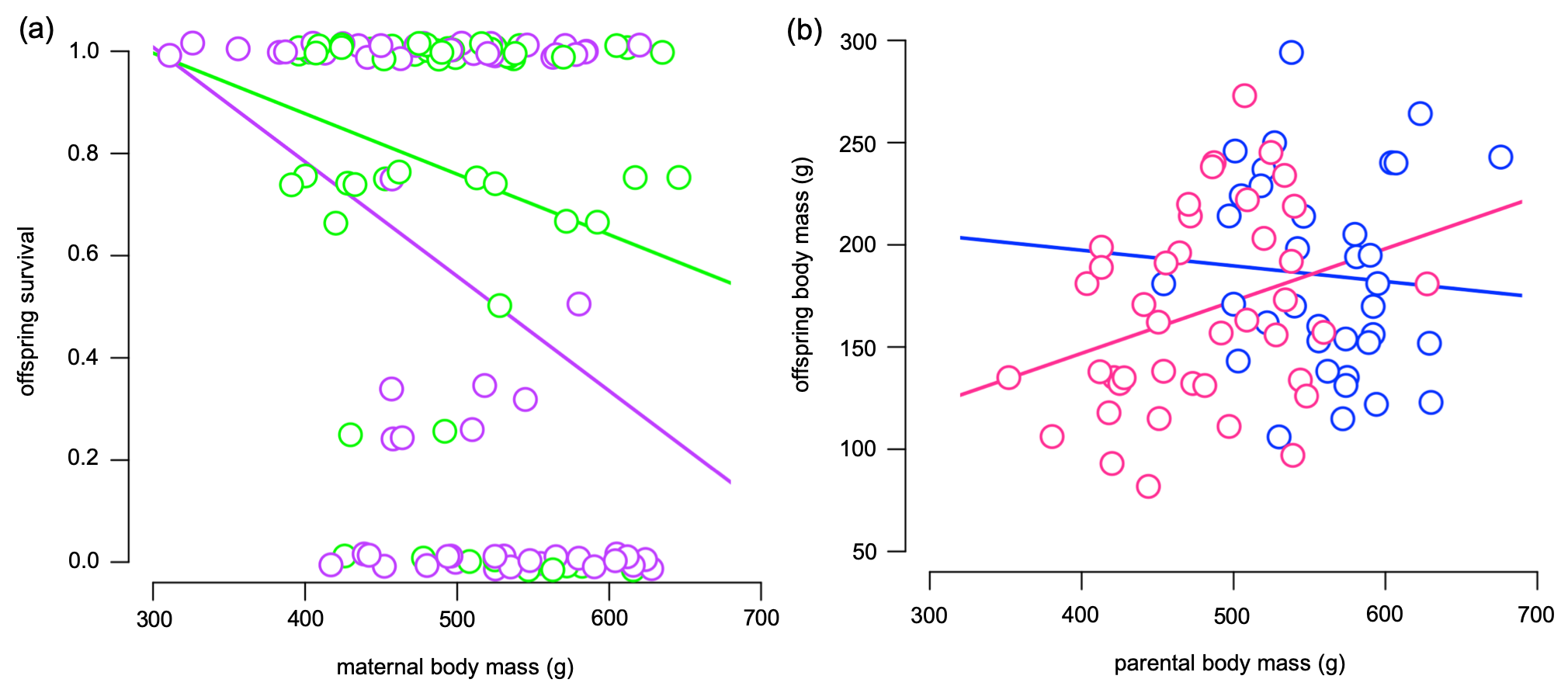
**

(*a*) A significant effect of maternal body mass on offspring survival, with offspring more likely to survive when born to heavier mothers, and a non-significant trend of greater survival among offspring born to mothers sourced from the wild (green) compared to captive-bred mothers (purple). Data displayed are the proportion of offspring within each litter that survived to ~200 days of age. (*b*) A significant interaction between mother (pink) and father (blue) body mass on offspring mass; larger mothers, but smaller fathers, produced larger offspring in the captive numbat population. Data displayed are the average offspring weights for mothers and fathers across multiple litters, with the restriction of offspring weighed at an age of between 200 and 300 days.

Offspring sex ratios (OSR) of captive-bred (*n* = 23) and wild sourced (*n* = 24) mothers that contributed litters to the Perth Zoo numbat population (1993 – 2021):


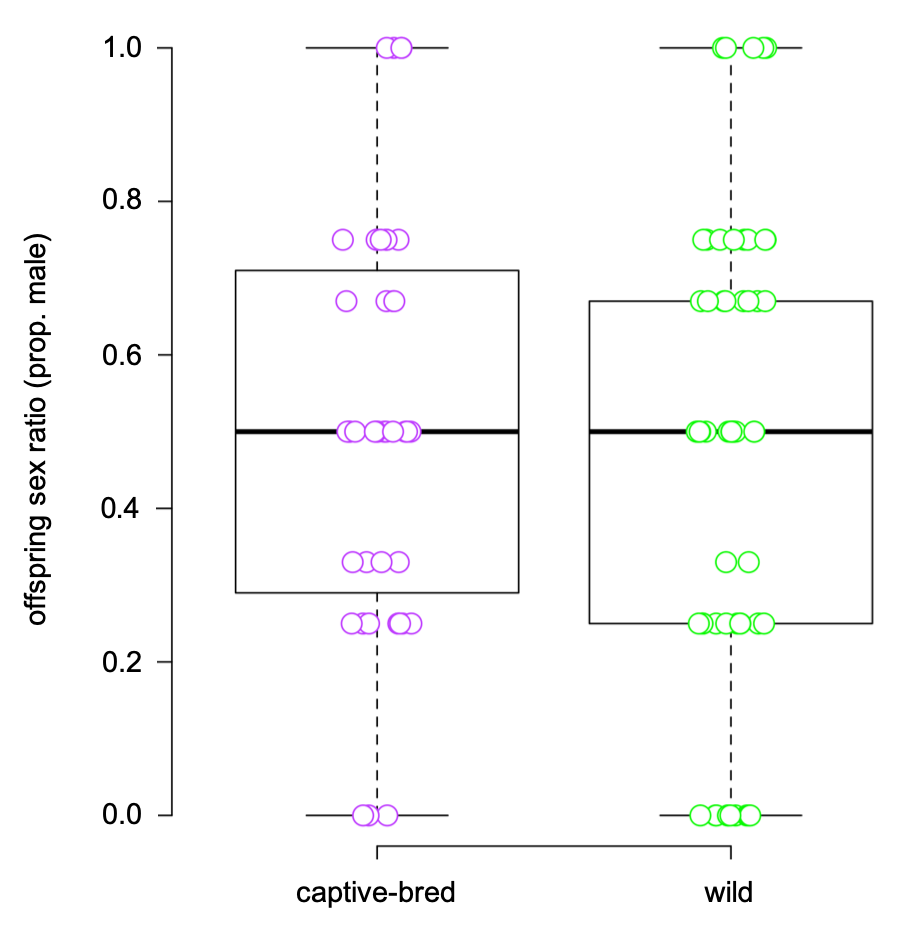


OSRs did not differ from parity (captive-bred: *Z*-value = 0.336, *p* = 0.737; wild: *Z*-value = 0.935, *p* = 0.350).
